# Photoacoustic-ultrasound endoscopy for assessment of rectal cancer treatment response: A prospective study with T2-weighted MRI radiomics comparison^☆^

**DOI:** 10.1016/j.pacs.2026.100840

**Published:** 2026-05-16

**Authors:** Haolin Nie, Sanskar Thakur, Anup Shetty, Lukai Wang, Md. Iqbal Hossain, Sitai Kou, Ahmed Eltahir, Jared Yee, Aaron Luo, Steve R. Hunt, Matthew G. Mutch, William C. Chapman, Quing Zhu

**Affiliations:** a Department of Biomedical Engineering, Washington University in St. Louis, St. Louis, MO 63130, USA; b Imaging Science Program, Washington University in St. Louis, St. Louis, MO 63130, USA; c Mallinckrodt Institute of Radiology, Washington University School of Medicine, St. Louis, MO 63110, USA; d Department of Surgery, Washington University School of Medicine, St. Louis, MO 63110, USA

**Keywords:** Acoustic resolution photoacoustic microscopy, Ultrasound, MRI radiomic models, Deep-learning, Rectal cancer treatment

## Abstract

Accurate identification of pathological complete response (pCR) following neoadjuvant chemoradiotherapy for locally advanced rectal cancer (LARC) is critical for non-operative "watch-and-wait" strategies. In this study, we evaluated whether co-registered acoustic resolution photoacoustic microscopy and ultrasound (ARPAM-US) endoscopy with deep learning could better identify pCR than T2-weighted MRI radiomics models. A pretrained ResNet50 model utilizing ARPAM-US B-scans was developed on a prospective cohort (n = 25) to predict pCR, based on surgical histopathological assessment, using 5-fold cross-validation. MRI radiomics models were trained on a retrospective cohort (n = 119). Performances between ARPAM-US ResNet50 and MRI radiomics models were compared. The ARPAM-US model achieved promising diagnostic performance (AUC 0.956, 95% CI 0.912–1.000), distinguishing normalized vascular architecture in complete responders. MRI radiomics models demonstrated degraded prospective generalization. While limited responders precluded definitive head-to-head comparison, deep learning-enhanced ARPAM-US endoscopy demonstrates promising diagnostic accuracy for assessing rectal cancer treatment response. Larger prospective studies are needed to validate these findings.

## 1 Introduction

Colorectal cancer (CRC) remains a significant public health challenge, ranking as the third most common cancer and the second leading cause of cancer-related deaths in the United States. In 2026 alone, an estimated 158,850 new CRC cases and 55,230 deaths are expected [1], with a concerning rise in incidence among adults younger than 55 years [2]. For locally advanced rectal cancer (LARC), the standard of care (SoC) involves neoadjuvant chemoradiotherapy (nCRT) followed by total mesorectal excision (TME). While TME is effective for local control, it is associated with substantial morbidity, including permanent ostomies, bowel dysfunction, and reduced quality of life [3], [4]. Conversely, neoadjuvant therapy can induce a pathologic complete response (pCR) in 10–35% of patients [5], [6]. To spare these patients from unnecessary surgery, the “watch-and-wait” strategy was introduced, wherein surgery is deferred in favor of rigorous surveillance [7], [8], [9].

The success of the watch-and-wait approach relies almost entirely on the accurate assessment of treatment response. Clinicians must reliably distinguish between scar tissue (fibrosis) and residual viable tumor. Neoadjuvant therapy can induce fibrosis in both normal and cancerous tissue, further complicating this distinction. Currently, this assessment relies on digital rectal exam, white-light endoscopy, and magnetic resonance imaging (MRI) [10]. However, each modality has critical limitations. Digital rectal exam is limited to a few centimeters from the anal verge and suffer from operator dependence. Endoscopy is limited to the mucosal surface and cannot evaluate residual disease within the submucosa or deeper within the bowel wall. Standard clinical MRI interpretation, while excellent for staging, suffers from limited spatial resolution and difficulty in differentiating post-treatment fibrosis from viable tumor tissue [11], [12]. For post-neoadjuvant therapy restaging using conventional T2-weighted imaging (T2w), MRI has an overall mean sensitivity of 50.4% and mean specificity of 91.2%. For identifying T0 from T1–4 after neoadjuvant therapy, T2w showed a mean sensitivity of only 19.1% with specificity of 94.6% [13]. These figures are primarily based on T2w sequences. The addition of diffusion-weighted imaging (DWI) has since shown improved sensitivity for detecting residual tumor and reduced reader dependence [13], and DWI is increasingly considered part of the standard restaging protocol. Nevertheless, T2w MRI remains highly reader sensitive: experienced readers (≥5 years of rectal MR imaging) showed significantly better overall mean sensitivity than less experienced (<5 years rectal MR imaging) readers for restaging (70% vs 47.4%) [13]. Standard biopsy carries a false negative rate as high as 11% [14], highlighting the urgent need for a high-resolution, transmural imaging modality capable of a “virtual biopsy”.

MRI radiomics is an emerging translational field that extracts high-dimensional quantitative features, such as texture, shape, and intensity, to identify biological patterns invisible to the naked eye [15], [16]. In rectal cancer, MRI-based radiomics have demonstrated the ability to predict treatment outcomes, including pCR [17], [18], lymph node metastasis [19], and long term disease free survival [20]. MRI radiomics has shown good aggregate performance for predicting pCR after neoadjuvant therapy. A recent meta-analysis of 35 studies (n = 10,543) reported a pooled AUC of 0.87 (95% CI 0.84–0.89) for pCR detection. Subgroup analyses showed that 3 T imaging outperformed 1.5 T (AUC 0.90 vs. 0.82, p < 0.001), and that Asian populations demonstrated higher diagnostic performance than non-Asian populations (AUC 0.90 vs. 0.80, p < 0.001) [21]. However, the 95% confidence intervals indicated substantial variability, with mean lower and upper bounds of 0.745 ± 0.145 and 0.927 ± 0.087, respectively. Another meta-analysis on 35 MRI-based radiomics studies (n = 9696) reported pooled sensitivity and specificity of 83% and 82% respectively, with a pooled AUC of 0.91 [22]. However, the same analysis found substantial heterogeneity related to factors such as MRI sequence selection, field strength, contouring, and feature-processing workflows, underscoring persistent challenges in reproducibility and external generalization. The same analysis also reported that radiomics with deep-learning showed higher sensitivity and specificity compared with conventional radiomics, however, the differences were not statistically significant. Our reported MRI study of two cohorts also experienced large variations in image acquisition parameters (e.g. field strength, pixel spacing, and scanner manufacturer) (see Table A.1). Nevertheless, MRI radiomics represents an advanced computational approach currently being extensively investigated for rectal cancer response assessment.

Photoacoustic (PA) imaging is uniquely positioned to address this diagnostic gap. By combining the tissue morphology contrast of ultrasound (US) with the optical contrast of PA imaging, PA imaging has shown to image vascular architecture and hemoglobin oxygen saturation, which are key functional biomarkers for tumor viability [23], [24]. PA has shown promise in various oncologic diagnostic applications, such as thyroid [25], ovarian [26], [27], and breast cancers [28], [29], [30]. PA has also been investigated in determining radiotherapy treatment response in head and neck tumors [31].

Utilizing PA imaging to assess rectal cancer treatment response has been extensively investigated by our group. We previously advanced acoustic-resolution photoacoustic microscopy (ARPAM) from benchtop feasibility studies [32] to a co-registered pilot endoscopic evaluation in patients [33]. More recently, we have developed a portable system designed for patient studies at the point-of-care [34]. Despite these advances, two critical questions remain. First, can ARPAM-US data be analyzed robustly without manual processing? Previous studies relied on manual selection of region-of-interest (ROI), whereas clinical translation requires automated assessment. Second, how does ARPAM-US compare to, or complement, advanced MRI analysis? While MRI radiomics and deep learning have been explored to predict neoadjuvant treatment response, it remains unclear if adding MRI data to high-resolution endoscopic ARPAM-US data would improve diagnostic accuracy for local assessment of rectal cancer.

To address these questions, we report a deep learning–based ARPAM-US endoscopy system in a prospective cohort of LARC patients following neoadjuvant treatment. We implemented a simplified deep learning pipeline to predict pCR and conducted exploratory comparison with T2-weighted MRI-based radiomics models. Our goal is to determine whether coregistered ARPAM-US functional and structural endoscopy alone is sufficient for assessing local treatment response, or if multimodal fusion with MRI offers superior predictive value.

To the best of our knowledge, this is the first prospective study to evaluate the predictive performance of ARPAM-US endoscopy for pCR and to investigate its correlation with T2-weighted radiomic MRI models. It is also the first investigation of ARPAM-US endoscopy relative to the current SoC MRI.

## 2 Methods

### 2.1 Coregistered PAM-US imaging system

**Fig. 1:**
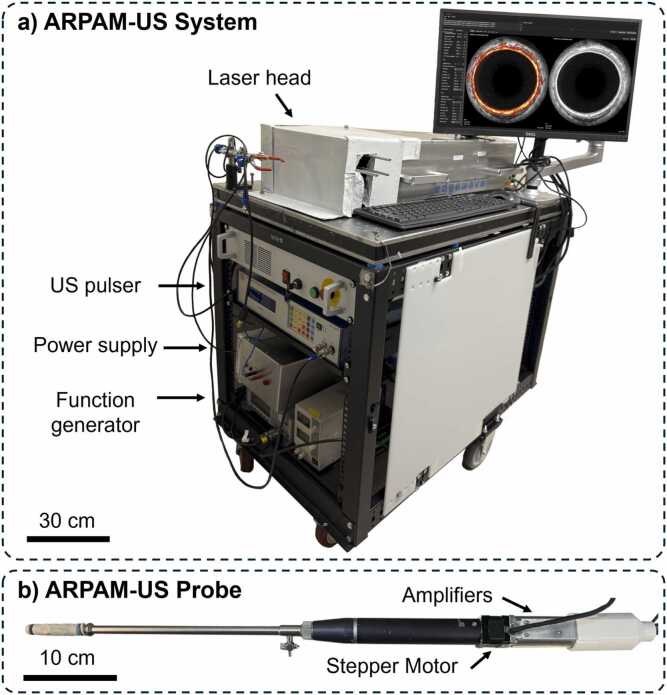
ARPAM-US system (a) and probe (b).

The portable ARPAM-US endoscopic system was developed and optimized as described in our prior work [34]. The current system is shown in Fig. 1**a**. Optical excitation was achieved using a Q-switched Nd:YAG laser (1064 nm, 9 ns pulse width, 1 kHz repetition rate; Edgewave, Germany). Light was coupled via a multimode fiber through the center of a custom ring-shaped, focused ultrasound transducer (10 mm OD, 1.8 mm ID, 25 MHz central frequency, 115% bandwidth, 15 mm focus; Capistrano Labs, CA, USA). The diameter of the imaging head was 13.6 mm and the theoretical fluence at the probe tip was up to 26 mJ/cm2, within the ANSI safety limit for skin exposure at 1064 nm [34]. The system achieved axial and lateral resolutions of 72 µm and 116 µm for ARPAM and 35 µm and 122 µm for US, respectively. The signal-to-noise ratio (SNR) was approximately 50 dB for PA and 30 dB for US in the near and focal fields [34]. A mechanical rotation of the probe head at 1 Hz via a stepper motor enabled 360-degree side-view B-mode imaging (Fig. 1**b**). Laser pulsing, ultrasound transmission, and data acquisition were synchronized by a function generator and controlled by a custom C+ + interface (ArpamGui [35]), which enabled parameter tuning, real-time visualization, and intraoperative image annotation.

### 2.2 ARPAM-US cohort and imaging protocol

**Fig. 2:**
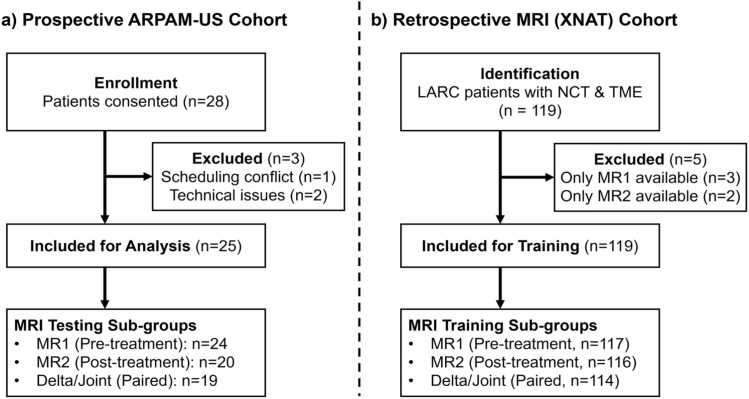
Patient enrollment information for (a) the prospective ARPAM-US cohort and (b) the retrospective MRI XNAT cohort.

This prospective study was approved by the Washington University School of Medicine institutional review board (IRB, clinical.trial.gov NCT04339374). Patients with biopsy-proven rectal cancer of any stage, located within 15 cm of the anal verge and scheduled for surgical resection were eligible for inclusion. Between May 2024 and August 2025, 28 consecutive patients provided written informed consent. Three patients were excluded from the final analysis: one due to scheduling conflict preventing imaging prior to surgery and two due to technical malfunctions during image acquisition. The APRAM-US cohort enrollment is summarized in Fig. 2**a**.

ARPAM-US imaging was conducted in the operating room after induction of general anesthesia. The study surgeon used a standard white light proctoscope to locate the approximate tumor bed location. Then, the ARPAM-US probe was inserted through the proctoscope and advanced to pass the estimated tumor bed location. Co-registered US and PA B-scans were acquired at a constant speed as the probe was manually withdrawn from the rectum, capturing multiple frames of both the tumor bed and adjacent normal tissue. Each imaging session lasted approximately 3–5 min depending on the tumor bed relative to the anal verge. The imaging probe was then removed. The surgeon then performed standard-of-care surgical resection. All resected specimens were evaluated by attending pathologists according to the AJCC 8th edition TNM staging system and categorized by tumor regression grade (TRG). Pathologic complete response (pCR) was defined as the absence of viable tumor cells in both the primary site and the regional lymph nodes (ypT0N0 and TRG 0). When available, pre-treatment (before nCRT) and post-treatment (after nCRT but before surgery) MRI images were acquired for analysis. This patient cohort is hereafter referred to as the ARPAM-US cohort.

Tumor bed regions were identified intraoperatively by the study surgeon and annotated within ArpamGui immediately following acquisition. No significant operator dependence was observed in the acquired data and multiple scans were selected for each patient to account for any unobserved variations. For each patient, approximately 16 B-scans were selected for analysis: eight representing the tumor bed and eight representing the normal rectum (either proximal or distal to the tumor). These ‘representative’ scans were chosen based on three pre-defined quality criteria: 1. Full 360-degree visualization of the rectal wall circumference, 2. High SNR in both the US and PA channels to ensure clear layer differentiation, and 3. Alignment with intraoperative annotations provided by the study surgeon immediately following acquisition. To minimize selection bias, scans were selected sequentially from the center of the annotated tumor bed region rather than based on the visual intensity of the vascular signal.

### 2.3 ARPAM-US deep learning feature extraction

**Fig. 3:**
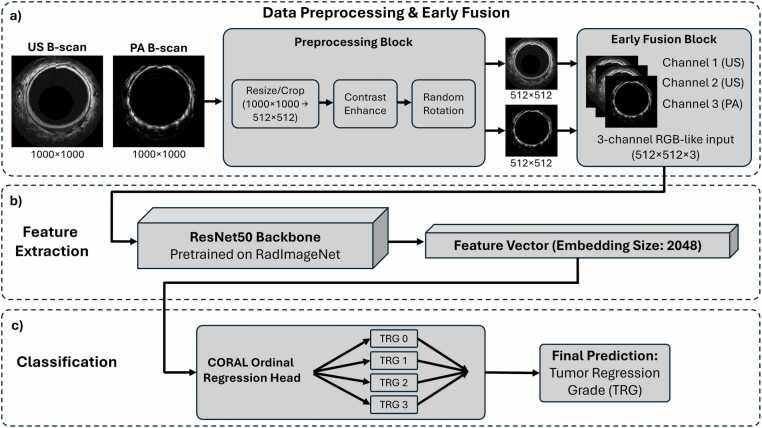
ARPAM-US deep learning pipeline. (a) Image preprocessing and early fusion. (b) Feature extraction using a ResNet50 backbone. (c) Ordinal regression using CORAL.

Whole co-registered PA and US B-scans served as the primary model inputs. To standardize input dimensions and reduce computational complexity, scans were resized-cropped from their original 1000×1000 pixel resolution to 512×512 pixels. The original dimentions already exceed the system's acoustic resolution (72 µm axial), so the down sampled pixel pitch remains on the order of the physical resolution limit, and mesoscale vascular patterns (vessel density, architectural disruption, wall layer continuity) relevant to treatment response assessment are preserved. Preprocessing steps included contrast enhancement and random rotation for data augmentation, and pixel value rescaling between [0, 1] (Fig. 3**a**).

We implemented a ResNet50 backbone for ordinal classification of T-stage and TRG (Fig. 3**b**). To leverage medical-domain transfer learning, the model was initialized with weights pretrained on the RadImageNet dataset [36]. Because these weights require 3-channel input, we employed a specific channel-mapping strategy: for multimodal ARPAM-US models, two channels were assigned to the US B-scan and one to the PA B-scan. For single modality models (US-only and PA-only), the respective B-scans were duplicated across all three channels. The entire ResNet50 backbone was unfrozen, allowing the pretrained weights to be fine-tuned specifically for the unique contrast and features of ARPAM-US endoscopy data. The network was optimized using the Consistent Rank Logits (CORAL) framework [37] to ensure ordinal consistency across the TRG and T-stage spectrum. Training was implemented in PyTorch using the CORAL loss function, a batch size of 16, the AdamW optimizer (learning rate $1\times 10^{-3}$, and one-cycle learning rate scheduler (Fig. 3**c**). Training for each split took approximately 5 min on a Nvidia GeForce RTX 4070 Super.

To determine final diagnostic outputs from the multiple B-scans acquired per patient (∼8 per tumor bed and ∼8 per normal region), a multi-step ensemble inference strategy was employed. Because the models were trained using 5-fold cross-validation, inference for each B-scan was performed by an ensemble of the five resulting models. We took the CORAL head’s predicted probability for TRG 0 as the binary probability for pCR at the individual scan level. The final patient-level prediction was then derived by taking a majority vote across the ensemble’s predictions for all representative B-scans associated with that patient.

### 2.4 MRI cohort and exploratory analysis

To contextualize the performance of ARPAM-US endoscopy and explore the potential complementarity of multimodal imaging, we conducted an exploratory evaluation of MRI radiomics. Given the small sample size of the prospective ARPAM cohort relative to the high dimensionality of radiomics feature spaces, we recognized that training robust MRI radiomics models de novo on this cohort would be underpowered. Therefore, we identified a retrospective cohort of rectal cancer patients to develop radiomics models, then tested their ability to generalize to the prospective setting. Using the models derived from this cohort, we tested 1) MRI radiomics model generalizability across retrospective and prospective cohorts and 2) how MRI features might complement ARPAM-US endoscopic data.

#### 2.4.1 Retrospective MRI (XNAT) cohort

A retrospective cohort of 119 patients with rectal cancer treated between January 2020 and March 2024 was identified from institutional archives. Inclusion criteria were: biopsy-proven rectal cancer of any stage, completion of neoadjuvant treatment, surgical resection and pathologic assessment of the rectal specimen at Washington University School of Medicine, and availability of axial T2-weighted MRI sequences at diagnosis and after conclusion of neoadjuvant treatment. Radiomics analysis was restricted to axial T2-weighted images because this sequence was the most consistently available across both cohorts and permitted standardized segmentation and feature extraction for cross-cohort validation. DWI was not included because its availability and acquisition characteristics were insufficiently uniform across the retrospective and prospective datasets for robust harmonized radiomics analysis. To enable longitudinal radiomics analysis, 5 patients who only underwent a single MRI were excluded. The resulting MRI cohort included 114 patients with paired pre- and post-treatment MRIs. For single-timepoint modeling, features were extracted from all available scans (MR1 n = 117, MR2 n = 116), while paired-timepoint modeling used the paired subset (n = 114). The patient recruitment flow chart is summarized in Fig. 2**b**. Patient characteristics are summarized in Table 2. MRI acquisition parameters, which varied across the retrospective collection period, are detailed in Appendix A, Table A.1.

#### 2.4.2 MRI radiomics feature extraction

Primary tumor beds were segmented on axial T2-weighted MR images by a subspecialty-trained, abdominal radiologist with 10 years of post-training experience using the “XNAT” imaging platform, named as XNAT cohort in the following text [38], correlating with diffusion-weighted and post-contrast T1-weighted imaging where available. For post-treatment scans where no viable tumor was visible, the original tumor bed location was segmented. Images were resampled to uniform 0.75 × 0.75 mm in-plane resolution to standardize for acquisition differences; slice thickness was preserved to avoid through-plane interpolation artifacts.

**Fig. 4:**
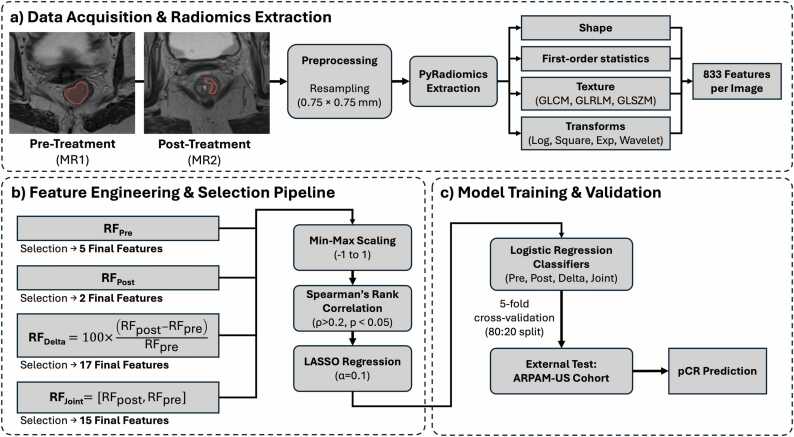
MRI radiomics pipeline for exploratory comparison. (a) Feature extraction from T2-weighted images using PyRadiomics. (b) Feature selection performed on the full XNAT cohort to maximize stability for external validation. (c) Logistic regression models trained via 5-fold cross-validation on XNAT, then tested on prospective ARPAM cohort MRI data.

Radiomics features were extracted using PyRadiomics [39], yielding 833 features per image: 14 shape descriptors, 18 first-order statistics, and 73 texture features (GLCM, GLRLM, GLSZM), plus derivatives from mathematical transforms (square, exponential, logarithm, and 8 wavelet decompositions). Fig. 4**a** shows the radiomics feature extraction pipeline.

For patients with paired MRI, we generated two derived feature sets: Delta-radiomics (${\text{RF}}_{\text{delta}}=100\times \left({\text{RF}}_{\text{post}}-{\text{RF}}_{\text{pre}}\right)/{\text{RF}}_{\text{pre}}$) [17], representing the percentage change in features following neoadjuvant treatment, where ${\text{RF}}_{\text{pre}}$ and ${\text{RF}}_{\text{post}}$ are the pre- and post-treatment radiomic features respectively, and Joint-radiomics (${\text{RF}}_{\text{joint}}=[{\text{RF}}_{\text{post}},\;{\text{RF}}_{\text{pre}}\text{]}$), created by the concatenation of pre- and post-treatment features. This resulted in four feature groups for modeling: ${\text{RF}}_{\text{pre}}$, ${\text{RF}}_{\text{post}}$, ${\text{RF}}_{\text{delta}}$, and ${\text{RF}}_{\text{joint}}$.

#### 2.4.3 Radiomic feature selection and model training on XNAT cohort

Radiomic feature selection was performed on the full XNAT cohort to maximize stability of the selected feature sets, which would subsequently be applied to the prospective ARPAM cohort for external validation. We applied a three-step pipeline (Fig. 4**b**): First, min-max scaling was performed to scale each value between −1 and 1. Second, Spearman’s rank correlation was calculated between each feature and pCR status; features with a correlation > 0.2 and p-value < 0.05 were retained. Finally, LASSO regression (alpha = 0.1) was used to remove zero-weighted features. This yielded final feature sets of ${\text{RF}}_{\text{pre}}$ (5 features), ${\text{RF}}_{\text{post}}$ (2 features), ${\text{RF}}_{\text{delta}}$ (17 features), and ${\text{RF}}_{\text{joint}}$ (5 features). Selected features are detailed in **Appendix A, Tables A.2-A.3**.

For each feature group, logistic regression classifiers were trained using 5-fold cross-validation on the XNAT cohort (80:20 train-validation split per fold, Fig. 4**c**) to estimate internal validation performance. These trained models were then applied to the prospective ARPAM cohort MRI data as an external test set.

#### 2.4.4 Exploratory radiomic and deep learning feature extraction and model training on XNAT cohort

In this section, we further explored a fusion strategy that combines MRI radiomic features selected in Section 2.4.3 and deep learning features. The study was intended to confirm the impact of domain shift experienced in radiomic models. A ResNet-18 model pretrained on ImageNet and fine-tuned on the XNAT cohort was used to extract deep learning features. Features from the penultimate layer of ResNet-18 were concatenated with selected radiomic features, forming a fused vector that captures both hierarchical image representations and morphological descriptors. Prior to concatenation, features were harmonized using log transformation (for skewed distributions) and quantile normalization across cohorts.

A support vector machine (SVM) with a radial basis function (RBF) kernel was trained from the fused feature vectors of XNAT cohort. Stability-based feature selection was then applied via 100 bootstrap iterations of mutual information ranking, retaining features selected in > 50% of resamples (n = 45 features). SVM training employed an imbalanced-learning pipeline with SMOTE-Tomek resampling confined within each cross-validation fold to prevent data leakage. Hyperparameters (C, Gamma) were optimized using nested cross-validation, and the final model was evaluated on an independent prospective ARPAM-US cohort.

### 2.5 Exploratory ARPAM-US-MRI feature fusion

To explore whether macroscopic MRI radiomics might complement high-resolution endoscopic ARPAM-US data, we conducted exploratory late-fusion experiments. The selected radiomic feature vectors (${\text{RF}}_{\text{pre}}$, ${\text{RF}}_{\text{post}}$, ${\text{RF}}_{\text{delta}}$, and ${\text{RF}}_{\mathrm{joint}}$) from Section 2.4.3 were concatenated with deep learning features extracted from the ARPAM-US ResNet50 backbone before the final CORAL classification layer.

Given the small prospective sample size and the instability observed in both MRI radiomics and fusion experiments, these results are presented as exploratory findings to inform future multimodal study design rather than as definitive evidence for or against fusion strategies.

### 2.6 Statistical analysis

Model performance was evaluated using the area under the receiver operating characteristic curve (AUC) as the primary metric. For ARPAM-US models, 5-fold cross-validation was performed at the patient level, with predictions aggregated using majority voting across B-scans per patient. Mean AUC and 95% confidence intervals across folds were calculated using the Student’s t-distribution (two-tailed, α=0.05).

For MRI radiomics, internal validation using 5-fold cross-validation on the XNAT cohort, while external validation applied the trained models to the prospective ARPAM cohort. Given the exploratory nature of the MRI analysis and the small size of the prospective cohort, we did not perform formal statistical hypothesis testing comparing ARPAM-US and MRI model performance.

All analyses were performed using Python 3.12.9 with PyTorch 2.9.0, scikit-learn 1.7.2 and PyRadiomics 3.0.1. Code for the ARPAM-US acquisition, image reconstruction, and image annotation interface (ArpamGui) is available at https://github.com/OpticalUltrasoundImaging/ArpamGui.

## 3 Results

### 3.1 ARPAM-US patients

The final ARPAM-US cohort consisted of 25 patients (Fig. 2**a**). Demographic and pathological characteristics are detailed in Table 1. Pathological complete response (pCR, ypT0N0) was confirmed in 3 patients (12%), while the remaining 22 patients (88%) exhibited partial response or non-response (TRG 1–3) (Table 2).

**Table 1:** Prospective *In vivo* ARPAM-US patient characteristics.

**Characteristic**	**Value**
Age, mean (range)	62 (43−83)
**Sex**	
Male	18 (72%)
Female	7 (28%)
**Post-treatment T-stage**	
T0	4 (16%)
T2	10 (40%)
T3	8 (32%)
T4	3 (12%)
**Tumor Regression Grade (TRG)**	
TRG 0 (Pathologic Complete Response,pCR)	3 (12%)
TRG 1 (Near Complete Response)	3 (12%)
TRG 2 (Partial Response)	11 (44%)
TRG 3 (Poor/No Response)	5 (20%)
No Treatment (Staging only)	3 (12%)

**Table 2:** Retrospective MRI patient characteristics.

**Characteristic**	**Value**
Age, mean (range)	58 (24−83)
**Sex**	
Male	75 (63%)
Female	43 (36%)
**Pre-treatment T-stage (MR1)**	
T1	1
T2	14
T3	64
T4	39
**Post-treatment T-stage (MR2)**	
T0	13
T1	5
T2	34
T3	50
T4	16
**Outcome Metrics**	
pCR (TRG 0)	17 (15%)*
Residual tumor (TRG 1–3)	97 (85%)*

* Derived from final training subset (n=114 with paired MRI).

### 3.2 Qualitative imaging findings

**Fig. 5:**
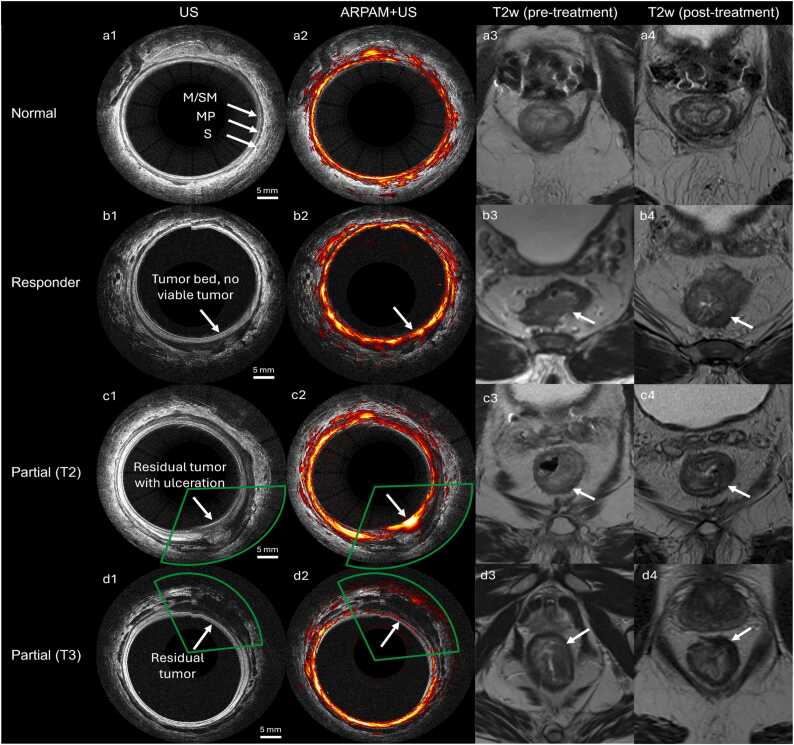
Representative ARPAM-US B-scans and pre- and post-treatment T2w MRI across response categories. Normal rectum shows distinct wall layers on ultrasound (a1) and dense mucosal/submucosal vasculature on photoacoustic imaging (a2). In complete responders, photoacoustic imaging demonstrates restored vascular patterns (b2) in the treated tumor bed. Contrarily, partial responders exhibit disrupted vasculature (c2,d2) and ulceration (c2) on photoacoustic imaging; Notably, the disrupted vascular pattern at the 5-o’clock position (c2) corresponds to the sudden structural change seen on ultrasound (c1). However, these patterns are not differentiated on post treatment MRI (b4, c4, d4), where partial responders showed persistent intermediate T2 signal along the tumor bed without significant change from the pre-treatment exam.

While detailed microvascular architecture is not observed in the images, correlation of PA signal and vascularity in tumor bed was observed, consistent with our previously published work [33], [34], [40]. Visual inspection of representative B-scans illustrates the imaging features observed across response categories (Fig. 5). Normal rectum (Fig. 5**a**) displayed intact, continuous layered structures in the ultrasound channel and uniform, regular vascular signals in the photoacoustic channel. In patients with complete response (pCR, Fig. 5**b**), we observed a treated tumor bed with fibrosis in the ultrasound channel and a normalized vascular architecture similar to healthy rectum in the co-registered photoacoustic channel. For partial and non-responders (Fig. 5 **cd**), scans showed distinct pathological features, where ultrasound showed irregular wall thickening or disrupted layers, while the photoacoustic channel highlighted persistent, high-intensity signal at the periphery of the tumor bed indicative of tumor associated angiogenesis, ulceration, and disorganized microvasculature that remained despite neoadjuvant therapy. Notably, for partial responders with ulceration (Fig. 5**c2**), we observe strong surface PA signals corresponding to bleeding, but the signal intensity and shape are distinct from the PA signal of normalized microvasculature (Fig. 5**a2b2**).

For MRI findings, pre-treatment scans typically showed moderately hyperintense tumor on T2-weighted imaging with loss of normal rectal wall stratification. Post-treatment T2-weighted images of complete responders demonstrated either normalized rectal wall architecture or dense fibrosis presenting as low T2 signal fibrosis at the tumor bed. However, in many treated tumor beds the appearance is mixed, with varying contributions from fibrosis, edema, inflammation and potentially residual viable tumor; in these cases, intermediate T2 signal and irregular morphology can make distinction between benign post-treatment change challenging on T2-weighted images alone [41]. Notably, in several pathologically complete responders (e.g. Fig. 5b3–4), post-treatment T2-weighted MRI still described persistent mural abnormalities that could be interpreted as residual viable disease. This highlights a limitation of morphology-based T2 assessment for local tumor-bed evaluation and supports the value of complementary functional imaging approaches when complete response is being considered.

### 3.3 Diagnostic performance of ARPAM-US deep learning models

**Fig. 6:**
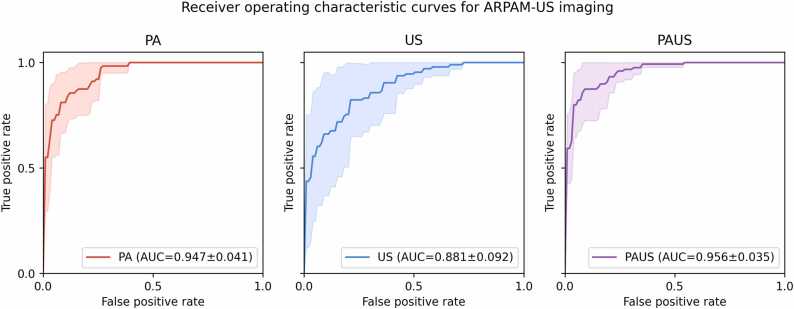
Receiver operating characteristic (ROC) curves for the PA, US, and PAUS fusion models.

We evaluated the performance of three deep learning models (US-only, PA-only, and ARPAM-US) using 5-fold cross-validation. The performance metrics are summarized in Fig. 6. The US-only model, relying on morphological features, achieved a mean validation AUC of 0.881 (95% CI: 0.767–0.995), though it demonstrated higher variability across folds compared to other approaches. The PA-only model, which captures functional information regarding hemoglobin distribution and vascular architecture, showed higher mean performance with a validation AUC of 0.947 (95% CI: 0.896–0.998). The early fusion (ARPAM-US) model, which combines the anatomical context of US with the functional specificity of PA, achieved a mean validation AUC of 0.956 (95% CI: 0.912–1.000).

### 3.4 MRI performance

Sample sizes varied across radiomics feature groups due to missing MRI timepoints: in the XNAT training cohort (n = 119), 2 patients lacked pre-treatment and 3 lacked post-treatment MRI, yielding RFpre = 117, RFpost = 116, and RFdelta/RFjoint = 114 (requiring both timepoints; Fig. 2b). In the prospective test cohort (n = 25), incomplete MRI availability (outside referrals, scheduling gaps) reduced samples to RFpre = 24, RFpost = 20, and RFdelta/RFjoint = 19 (Fig. 2a).

On internal validation, the best-performing model (RFjoint) achieved AUC 0.877, but dropped to 0.654 on prospective testing (Table 3). Similar degradation was observed for all feature groups (∆AUC −0.07 to −0.28), indicating that MRI radiomics models are highly sensitive to domain shifts from variations in acquisition parameters between cohorts.

**Table 3:** MRI radiomics model performance (rows 1–4) and combined radiomic and deep learning SVM model performance (last row): internal validation vs. prospective testing. Internal validation used 5-fold CV on XNAT cohort (n = 119). External cohort used prospective ARPAM cohort (n = 24 for RF_pre_, n = 20 for RF_post_, n = 19 for RF_delta_ and RF_joint_). AUC presented as mean and 95% CI.

**MRI Feature Group**	**XNAT Validation AUC** (Internal, n=114)	**ARPAM Testing AUC** (Prospective, n=25)	**Performance Drop** (Δ)
RF_pre_	0.777 (0.602–0.953)	0.543 (0.485–0.601)	-0.234
RF_post_	0.816 (0.664–0.969)	0.537 (0.481–0.593)	-0.279
RF_delta_	0.642 (0.463–0.820)	0.569 (0.454–0.684)	-0.073
RF_joint_	0.877 (0.795–0.958)	0.654 (0.625–0.684)	-0.223
RF_joint_ + DL	0.929 (0.894–0.977)	0.720 (0.671–0.739)	-0.209

In the next exploratory study of combining radiomic with deep learning features, the SVM model achieved a validation AUC of 0.929 on the retrospective cohort. However, performance decreased on the prospective ARPAM-US cohort (AUC = 0.720), yielding a generalization gap of ∼0.21. This drop also confirms the impact of domain shift: despite incorporating transferable deep learning features.

### 3.5 ARPAM-US-MRI multimodal fusion

We investigated whether adding MRI radiomics to the endoscopic data (late fusion) could improve predictive accuracy. The highest numerical performance was achieved by the PA+RFpost late fusion model (AUC 0.967, Table 4). However, the marginal improvement over the PAUS model was not statistically significant (p-value 0.524). This suggests that while MRI provides macroscopic staging information, the local, high-resolution vascular remodeling captured by ARPAM is more effective for determining local treatment response in the tumor bed.

**Table 4:** ARPAM-US-MRI fusion model performance matrix (modality + MRI features).

**MRI Feature Group**	**Endoscopic Modality (AUC [95% CI])**		
	**PA-only**	**US-only**	**PAUS (Early Fusion)**
Baseline (No MR)	0.947 (0.90–1.00)	0.881 (0.77–1.00)	**0.956 (0.91–1.00)**
*Late Fusion Models:*			
+ RF_pre_	0.963 (0.93–1.00)	0.874 (0.74–1.00)	0.933 (0.85–1.00)
+ RF_post_	**0.967 (0.92–1.00)**	0.905 (0.81–1.00)	**0.967 (0.89–1.00)**
+ RF_delta_	0.513 (0.14–0.89)	0.786 (0.58–0.99)	0.721 (0.46–0.98)
+ RF_joint_	**0.959 (0.91–1.00)**	0.821 (0.54–1.00)	0.803 (0.34–1.00)

## 4 Discussion

In this study, we demonstrated that portable ARPAM-US endoscopy can achieve high diagnostic accuracy for assessing treatment response in LARC patients following neoadjuvant treatment. Importantly, this performance was achieved in a challenging cohort with difficult-to-assess cases, where standard clinical evaluation failed to confidently exclude residual disease, as all patients proceeded to surgery.

### 4.1 ARPAM-US technical advances and performance drivers

The second-generation portable ARPAM-US system provided reliable, high-quality imaging suitable for advanced computational analysis in the clinical setting. A key advancement over prior feasibility studies [33], [40] is the simplification of the deep learning pipeline. While Lin et al. [40] automated ROI generation via a sliding window approach with a DenseNet backbone, this operated on local patches and required window-size tuning. The ResNet50-based model instead takes whole B-scans as input, preserving global spatial context such as circumferential wall continuity. This eliminates the sliding window preprocessing step and leverages RadImageNet pretrained weights [36] for medical-domain transfer learning. The comparable diagnostic performance (0.956 vs. 0.968) with a simpler, more clinically translatable pipeline supports this design choice.

Our comparative analysis of imaging modalities revealed that photoacoustic imaging provides the primary discriminative signal for pCR assessment. The PAM-only model achieved AUC 0.947 (95% CI: 0.896–0.998), substantially outperforming US-only (AUC 0.881, 95% CI: 0.767–0.995), with early fusion providing modest additional benefit (AUC 0.956, 95% CI: 0.912–1.000). This hierarchy aligns with biological expectations that US contrast relies on acoustic impedance mismatches, which are often confounded by post-treatment fibrosis and inflammation [11], [12], making it difficult to distinguish scar tissue from residual tumor. Conversely, PAM imaging directly maps hemoglobin distribution at microscopic resolution, providing a functional readout of vascular remodeling. Complete responders exhibited normal vascular architecture similar to healthy rectum, while non-responders displayed persistent chaotic angiogenesis patterns characteristic of viable tumor [42]. This demonstrates that functional vascular signatures are more reliable biomarkers for pCR than morphological features alone. PA signal intensity is inherently proportional to hemoglobin concentration and thus serves as a measure of microvascular density (MVD) [42]. The deep learning model implicitly captures these MVD-related spatial patterns from whole B-scans without requiring explicit quantification as a separate metric.

These results suggest that PA features provide the primary discriminative signal for pCR assessment. The improvement from early fusion (AUC 0.956 vs. PA-only 0.947) was not statistically significant (p > 0.05), indicating that the added benefit of US anatomical context cannot be distinguished from chance given the current sample size. However, US still offers practical value by facilitating identification of the anatomical tumor bed. Definitive conclusions about relative performance require validation in larger independent cohorts. The AUC for each individual fold in the 5-fold cross-validation is presented in Appendix A, Table A.4 to demonstrate model stability across data splits. All ARPAM-US data was acquired on the lab developed system with one of the two almost identical lab developed imaging probes and no significant difference was observed between the acquisition parameters. Since the reported data and the system are unique to our lab, we were unable to validate domain shift effects.

### 4.2 MRI analysis: exploratory findings and limitations

MRI T2-weighted radiomics models achieved high AUC 0.877 (95% CI 0.795–0.958) but degraded on prospective testing (AUC 0.654, 95% CI 0.625–0.684). The SVM model combining MRI radiomic and deep learning features achieved AUC 0.925 (95% CI 0.894–0.977), however, this decreased to 0.720 (95% CI 0.671–0.739). This performance drop should be attributed to data heterogeneity: different field strength (predominantly 1.5 T in XNAT vs. more 3.0 T in the prospective cohort), pixel spacing (0.643 ± 0.173 vs. 0.502 ± 0.191 mm), and scanner hardware (Table A.1), rather than to MRI as a modality. Our results are consistent with a recent meta-analysis [21] of 35 studies, confirming that acquisition parameters drive radiomics performance [43]. Retraining MRI models directly on the prospective cohort (n = 19 with paired MRI, 3 pCR) failed to achieve meaningful performance (AUC ≈ 0.5), confirming insufficient sample size for high-dimensional radiomics. By contrast, ARPAM-US models leveraged transfer learning from RadImageNet [36] to achieve reasonable performance despite the modest cohort size.

Exploratory late fusion (Table 4) produced inconsistent results; no formal statistical tests were performed, and numerically higher AUCs do not establish superiority over ARPAM-US alone. RFdelta features degraded all models, likely because the percentage-change computation amplifies noise when pre-treatment denominators are small and magnifies acquisition differences between timepoints. The inconsistent effect of RFjoint (improvement for PA-only but degradation for PAUS) likely reflects feature saturation: the PA-only model had capacity for complementary MRI features, whereas PAUS already combined structural and functional information, making additional features redundant and prone to overfitting in the small sample.

Several additional limitations of our MRI analysis should be mentioned. First, we utilized only axial T2-weighted images for radiomics model development because these were the most consistently available images across both cohorts with relative homogeneity of imaging technique. DWI was not used due to excess heterogeneity of acquisition characteristics. This choice improved cross-cohort consistency but likely underestimates the performance of contemporary MRI-based response assessment, where DWI is commonly incorporated and may improve complete-response detection [13]. Deep learning applied directly to MRI might better capture relevant patterns than hand-crafted radiomics, however, the domain shift between cohorts precludes definitive cross-modality comparison. Matched, prospectively acquired data under standardized protocols would be needed for further evaluation.

### 4.3 Study limitations

Beyond the MRI-specific limitations outlined above, several additional constraints warrant acknowledgment. First, the cohort size, 25 patients with approximately 400 selected B-scans, is limited. We compensated for this with transfer learning, pretraining the ResNet50 feature extractor on RadImageNet. Although consistent performance across cross-validation folds (Appendix A, Table A.4) supports model stability, ARPAM-US faces the same external validation challenge identified for MRI radiomics. However, ARPAM-US relies on direct optical absorption contrast from hemoglobin, a physical signal less likely to be confounded by acquisition-specific parameters than radiomics texture features, which are statistical summaries of pixel intensity distribution. Additionally, the deep learning features are more compact and task-specific than the hand-crafted radiomics features. Nevertheless, validation in independent patient cohorts remains essential prior to clinical translation. Second, all enrolled patients underwent surgery, yielding a dataset enriched with relatively few pCRs. The subsequent phase of the study will therefore recruit patients assessed in endoscopy suites, encompassing a broader spectrum of clinical responders undergoing “watch-and-wait” management. This design will enable evaluation across a more representative population of complete, partial, and non-responders. Importantly, the authors also acknowledge the ARPAM-US system limitation. Single wavelength systems are unable to evaluate oxygen saturation in the vasculature which can be a critical parameter in assessing tumor microenvironment. We expect to further improve the system and incorporate multi-wavelength ARPAM-US imaging of rectal tumors in future work.

While our exploratory analysis suggests that ARPAM-US may offer advantages for local tumor bed assessment, the comparison is confounded by multiple factors: different training paradigms (cross-validation vs. external testing), use of only T2-weighted MRI, and insufficient sample size for evaluation. We emphasize that our findings suggest ARPAM-US is promising for local assessment of the tumor bed, but do not establish that it should replace MRI for comprehensive cancer restaging, where MRI’s larger field of view remains valuable for assessing mesorectal fascia involvement, lymph nodes, and extramural vascular invasion.

### 4.4 Future work

This proof-of-concept study establishes the feasibility of ARPAM-US endoscopy for rectal cancer restaging. Critical next steps include: a) larger prospective validation cohort with external validation; b) prospective evaluation comparing outcomes in patients managed with standard-of-care assessment vs. ARPAM-US augmented assessment. For MRI radiomics, future work will further explore deep learning approaches, incorporating DWI and other functional sequences, and implementing strategies to improve generalization across different scanner settings and hardware. Additionally, integrated models that combine deep learning and hand-crafted radiomic features extracted from the same endoscopic PA-US data, as recently demonstrated for breast cancer photoacoustic imaging [44], may offer a more stable alternative to external MRI fusion by avoiding the domain shift and resolution mismatch inherent in combining microscopic endoscopic data with macroscopic MRI texture features. For the ARPAM-US system, while we did not observe significant differences in data acquired by different surgeons, acquisition of 3D sequences for ARPAM-US imaging with manual pull-back is challenging. To address this we are working on developing an automated 3D acquisition probe capable of imaging the tumor region with automated helical motion [45].

## 5 Conclusion

We demonstrated that deep learning-enhanced ARPAM-US endoscopy can achieve high diagnostic accuracy for pCR assessment in a prospective cohort of rectal cancer patients undergoing surgery after nCRT (validation AUC 0.956, 95% CI: 0.912–1.000). The technique’s ability to directly visualize functional vascular remodeling at microscopic resolution offers potential advantages over current imaging modalities for local tumor bed evaluation. While exploratory comparison suggests that MRI T2-weighted radiomics without and will deep learning models suffer from poor generalization across cohorts, definitive comparative conclusions are limited by sample size and methodological constraints. External validation in larger, independent cohorts is essential to establish clinical utility and guide the integration of ARPAM-US endoscopy into watch-and-wait management protocols for rectal cancer.

## CRediT authorship contribution statement

**Mutch Matthew:** Writing – review & editing, Visualization, Validation, Supervision, Project administration, Methodology, Investigation, Funding acquisition, Data curation, Conceptualization. **Hunt Steven:** Writing – review & editing, Visualization, Validation, Supervision, Methodology, Investigation, Data curation, Conceptualization. **Aaron Luo:** Methodology, Investigation, Data curation. **Jared Yee:** Methodology, Investigation, Data curation. **Ahmed Eltahir:** Methodology, Investigation, Data curation. **Sitai Kou:** Investigation, Data curation. **Hossain Md Iqbal:** Validation, Methodology, Investigation, Formal analysis. **Lukai Wang:** Validation, Investigation, Data curation. **Anup Shetty:** Writing – review & editing, Visualization, Validation, Supervision, Software, Resources, Methodology, Investigation, Formal analysis, Data curation, Conceptualization. **Quing Zhu:** Writing – review & editing, Visualization, Validation, Supervision, Resources, Project administration, Methodology, Investigation, Funding acquisition, Formal analysis, Data curation, Conceptualization. **Sanskar Thakur:** Writing – review & editing, Writing – original draft, Visualization, Validation, Methodology, Investigation, Formal analysis, Data curation, Conceptualization. **Chapman Jr William:** Writing – review & editing, Visualization, Validation, Supervision, Resources, Project administration, Methodology, Investigation, Funding acquisition, Data curation, Conceptualization. **Haolin Nie:** Writing – review & editing, Writing – original draft, Visualization, Validation, Software, Methodology, Investigation, Formal analysis, Data curation, Conceptualization.

## Declaration of Competing Interest

The authors declare that they have no known competing financial interests or personal relationships that could have appeared to influence the work reported in this paper.

Given their role as Associate Editor, Quing Zhu had no involvement in the peer-review of this article and has no access to information regarding its peer-review. Full responsibility for the editorial process for this article was delegated to another journal editor.

## Figures and Tables

**Table A.1:** MRI Acquisition Parameters by Cohort

**Parameter**	**CNDA Cohort** (n=119, $N_{\mathrm{sessions}}=233$)	**ARPAM Cohort** (n=25, $N_{\mathrm{sessions}}=44$)
**Field Strength**		
3.0 T	n=44	n=17
1.5 T	n=197	n=27
0.55 T	n=1	n=0
**Scanner Manufacturer**		
Siemens	n=117	n=23
GE/Philips	n=2	n=2
**Pixel Spacing (mm)**	0.643±0.173	0.502±0.191
**Slice Thickness (mm)**	3.47±0.91	3.17±0.65
**Echo Time (TE, ms)**	105.5±7.3	98.3±10.0
**Repetition Time (TR, ms)**	5601.6±1994.2	5980.1±2042.6

**Table A.2:** MRI Radiomics Spearman Features

	**Features**	**Correlation**	**P-value**
**MR1**	wavelet-HH_glcm_ClusterShade	-2.57E-01	5.18E-03
	wavelet-HH_gldm_SmallDependenceLowGrayLevelEmphasis	2.24E-01	1.53E-02
	exponential_firstorder_Kurtosis	-2.68E-01	3.47E-03
	exponential_firstorder_Skewness	-2.42E-01	8.46E-03
	exponential_glcm_Idmn	-2.05E-01	2.70E-02
	exponential_ngtdm_Strength	-2.05E-01	2.63E-02
**MR2**	original_shape_Elongation	-3.24E-01	3.95E-04
	original_shape_Flatness	-2.62E-01	4.44E-03
	original_shape_MinorAxisLength	-2.09E-01	2.41E-02
**Delta**	original_firstorder_Maximum	2.23E-01	1.73E-02
	original_glrlm_LowGrayLevelRunEmphasis	2.13E-01	2.26E-02
	original_glrlm_RunPercentage	-2.34E-01	1.21E-02
	original_glrlm_ShortRunLowGrayLevelEmphasis	2.25E-01	1.60E-02
	original_gldm_LargeDependenceEmphasis	-2.26E-01	1.56E-02
	wavelet-LH_gldm_DependenceVariance	-2.03E-01	3.07E-02
	wavelet-LH_gldm_SmallDependenceLowGrayLevelEmphasis	2.16E-01	2.10E-02
	wavelet-HH_gldm_SmallDependenceLowGrayLevelEmphasis	2.01E-01	3.21E-02
	wavelet-LL_firstorder_Maximum	2.29E-01	1.45E-02
	wavelet-LL_glcm_Imc1	2.41E-01	9.76E-03
	wavelet-LL_glrlm_LowGrayLevelRunEmphasis	2.16E-01	2.10E-02
	wavelet-LL_glrlm_ShortRunLowGrayLevelEmphasis	2.27E-01	1.52E-02
	wavelet-LL_glszm_GrayLevelVariance	2.81E-01	2.50E-03
	wavelet-LL_glszm_LowGrayLevelZoneEmphasis	3.21E-01	5.02E-04
	wavelet-LL_glszm_SmallAreaLowGrayLevelEmphasis	2.99E-01	1.23E-03
	wavelet-LL_glszm_ZoneEntropy	2.68E-01	3.95E-03
	wavelet-LL_glszm_ZonePercentage	-2.18E-01	2.00E-02
	wavelet-LL_gldm_SmallDependenceLowGrayLevelEmphasis	2.87E-01	1.94E-03
	square_firstorder_Maximum	2.50E-01	7.23E-03
	square_firstorder_Range	2.50E-01	7.23E-03
	square_firstorder_Skewness	2.44E-01	8.76E-03
	square_glrlm_RunEntropy	2.09E-01	2.55E-02
	square_glszm_GrayLevelVariance	2.43E-01	9.25E-03
	square_glszm_SmallAreaHighGrayLevelEmphasis	3.01E-01	1.12E-03
	exponential_firstorder_Skewness	2.20E-01	1.86E-02
	exponential_glcm_JointEnergy	2.08E-01	2.67E-02
	exponential_glrlm_RunEntropy	2.04E-01	2.93E-02
	exponential_glszm_ZoneEntropy	2.08E-01	2.67E-02
	lbp-2D_glszm_SmallAreaEmphasis	2.36E-01	1.15E-02
**Joint**	mr1_wavelet-HH_glcm_ClusterShade	-2.54E-01	6.46E-03
	mr1_wavelet-HH_gldm_SmallDependenceLowGrayLevelEmphasis	2.21E-01	1.81E-02
	mr1_exponential_firstorder_Kurtosis	-2.64E-01	4.58E-03
	mr1_exponential_firstorder_Skewness	-2.37E-01	1.12E-02
	mr1_exponential_glcm_Idmn	-2.03E-01	3.07E-02
	mr2_original_shape_Elongation	-3.33E-01	2.90E-04
	mr2_original_shape_Flatness	-2.70E-01	3.61E-03
	mr2_original_shape_MinorAxisLength	-2.15E-01	2.15E-02

**Table A.3:** MRI Radiomics LASSO Features

	**Features**	**Weights**
**MR1**	wavelet-HH_glcm_ClusterShade	-3.88E-02
	wavelet-HH_gldm_SmallDependenceLowGrayLevelEmphasis	1.82E-02
	exponential_firstorder_Skewness	-1.66E-02
	exponential_glcm_Idmn	-1.04E-01
	exponential_ngtdm_Strength	-2.88E-02
**MR2**	original_shape_Elongation	-8.54E-02
	original_shape_Flatness	-1.58E-03
**Delta**	original_firstorder_Maximum	-2.09E-04
	original_glrlm_RunPercentage	-1.68E-05
	original_gldm_LargeDependenceEmphasis	-3.98E-05
	wavelet-LH_gldm_SmallDependenceLowGrayLevelEmphasis	1.16E-04
	wavelet-LL_firstorder_Maximum	1.54E-04
	wavelet-LL_glrlm_LowGrayLevelRunEmphasis	-2.76E-05
	wavelet-LL_glrlm_ShortRunLowGrayLevelEmphasis	-1.17E-05
	wavelet-LL_glszm_LowGrayLevelZoneEmphasis	1.82E-05
	wavelet-LL_glszm_SmallAreaLowGrayLevelEmphasis	3.82E-05
	wavelet-LL_glszm_ZoneEntropy	1.43E-05
	wavelet-LL_gldm_SmallDependenceLowGrayLevelEmphasis	2.46E-05
	square_firstorder_Maximum	2.33E-05
	square_glrlm_RunEntropy	4.90E-05
	square_glszm_GrayLevelVariance	5.28E-05
	exponential_firstorder_Skewness	-5.99E-05
	exponential_glcm_JointEnergy	1.17E-04
	exponential_glrlm_RunEntropy	-1.50E-05
**Joint**	mr1_wavelet-HH_glcm_ClusterShade	-2.86E-02
	mr1_wavelet-HH_gldm_SmallDependenceLowGrayLevelEmphasis	1.96E-02
	mr1_exponential_firstorder_Skewness	-2.47E-02
	mr1_exponential_glcm_Idmn	-9.94E-02
	mr2_original_shape_Elongation	-8.78E-02

**Table A.4:** Five-fold cross-validation performance (AUC) for the endoscopic modalities

**Fold Split**	**PA-only**	**US-only**	**PAUS (Early Fusion)**
Split 1	0.887	0.949	0.971
Split 2	0.991	0.996	0.979
Split 3	0.952	0.876	0.986
Split 4	0.928	0.795	0.899
Split 5	0.977	0.789	0.945
**Mean AUC**	**0.947**	**0.881**	**0.956**
Standard Deviation	0.041	0.092	0.035
95% Confidence Interval	[0.896, 0.998]	[0.767, 0.995]	[0.912, 1.000]

## Data Availability

Data will be made available on request.
